# Linguistic challenges of writing papers in English for scholarly publication: Perceptions of Chinese academics in science and engineering

**DOI:** 10.1371/journal.pone.0324760

**Published:** 2025-05-27

**Authors:** Yihua Zhang, Hiroyuki Eto, Jianlei Cui

**Affiliations:** 1 Graduate School of International Cultural Studies, Tohoku University, Sendai, Japan; 2 State Key Laboratory for Manufacturing Systems Engineering, Xi’an Jiaotong University, Xi’an, China; Kalasalingam Academy of Research and Education, INDIA

## Abstract

This study investigates the linguistic challenges encountered by Chinese academics in science and engineering when writing for English-language scholarly publications. Employing a mixed-methods approach, the research draws on survey responses from 732 participants and insights from semi-structured interviews with 13 interviewees. Sentence construction emerged as the most significant challenge, followed by issues with vocabulary selection, cohesive devices, coherence, and grammar, with notable variation across academic ranks. Common strategies to address these challenges include utilizing online tools, seeking peer support, and employing professional editing services. The findings offer actionable recommendations for tailored academic writing training, institutional support, and the integration of advanced technological tools, aiming to enhance publication success rates among non-native English-speaking scholars.

## Introduction

English has long been recognized as the *lingua franca* of global academia, particularly in research and scholarly publishing. This trend, initially identified by Swales [1] and Flowerdew [2], remains well-documented in contemporary literature [3–7]. In fields such as science and engineering, English dominates scholarly communication, with the vast majority of high-impact journals publishing primarily in English [8]. Indeed, over 95% of journals in the natural sciences and approximately 90% of those in the social sciences indexed by the Institute for Scientific Information (ISI) are published in English [9].

Non-native English-speaking scholars are increasingly required to produce research papers that adhere to the linguistic and rhetorical standards of English-medium journals [10]. This trend is especially prominent in China, where academic institutions and research organizations often mandate publication in internationally recognized journals for career advancement and institutional prestige.

In 2022, Chinese authors published over 735,600 papers, accounting for 28.9% of the global total. Despite the impressive increasing volume of publications, Chinese authors face higher rejection rates and more frequent requests for major revisions compared to their English-speaking counterparts [11–13]. Regardless of the originality of research or its alignment with journal scope, linguistic barriers are frequently cited as significant contributors to writing difficulties and manuscript rejection [14–17]. These challenges are more pronounced and burdensome than those encountered when writing in one's native language. A survey of nearly 600 Hong Kong Chinese academics found that over half perceived language-related issues as a major obstacle to publication [18]. This problem is particularly accurate for Chinese scholars, who often struggle with the nuances of academic English, including grammar, vocabulary, and stylistic conventions [19]. Jiang et al. [20], for example, surveyed 200 Chinese international students in Malaysia and found that inadequate grammar, limited vocabulary, and difficulty using accurate academic language significantly hindered their academic writing performance. Grammatical errors and lexical inaccuracies are especially common among non-native English writers, as consistently highlighted in numerous studies [21–27]. Particularly, the accurate application of academic vocabulary emerges as a persistent difficulty for non-native authors [28–31].

Different groups of academics share commonalities and differences in the challenges they encounter. Lin and Morrison [32] found that both graduate students and faculty at three local universities cited grammar and vocabulary as major writing challenges. Zhang and Liu [33] reported that many master's students struggled to construct coherent and grammatically correct sentences, often relying on simplistic sentences that may fail to convey the complexity of ideas required in academic discourse. Wu [34] observed that doctoral students faced issues with collocation and vague expressions. In Mu’s study [35], 34.2% of the research participants reported paper rejection due to writing issues such as redundancy, overly complex paragraphs, and grammatical errors.

Discourse-level challenges, including weak organization, sentence coherence, and intertextuality, are also critical challenges, as documented by Keong and Mussa [24] and Lin and Morrison [32]. Their findings emphasize the difficulty scholars face in achieving coherent and well-organized academic discourse, which is a key concern for both graduate students and faculty.

To address these linguistic challenges, various strategies have been proposed. Luo and Hyland [5,17,36] suggest that support from text mediators—language professionals with expertise in genre conventions—can significantly enhance the publication success for Chinese scholars. These professionals facilitate the transformation of manuscripts into publishable papers by discussing writing issues with authors. Additionally, training non-native English writers to become peer reviewers has also proven to be an effective strategy for improving academic writing [20,37,38]. This approach aligns with Hyland’s [3] assertion that peer feedback and academic collaboration play vital roles in the development of scholarly writing skills.

Another effective strategy involves using expert academic texts as models for writing. Both Lin and Morrison [32] and Hyland [3] found that analyzing well-written academic articles can help non-native English writers overcome linguistic challenges. Additionally, online tools like Google Translate are widely used among academics. While Lin and Morrison [32] caution that Google Translate may struggle with capturing meaning at the discourse level, Cancino and Panes [39] argue that it can still serve as a valuable pedagogical tool, provided that users are aware of its limitations and receive guidance on its effective application.

While much of the existing literature addresses the general challenges in the English academic writing faced by Chinese scholars, few studies specifically focus on science and engineering academics and compare challenges among different academic groups (e.g., university teachers vs. graduate students). Investigating these differences and their underlying causes contribute to the literature by highlighting the diversity of experiences within non-native English-speaking academics. This approach not only enriches our understanding of their writing challenges but also informs tailored interventions. Furthermore, identifying commonalities and differences assist policymakers and educators prioritize resources, ensuring equitable support across all career stages.

The research focuses on the following questions:

RQ1: What are the attitudes of Chinese academics toward writing papers in English for scholarly publication, and what is their current experience with publishing in English-language journals?

RQ2: What linguistic challenges do they encounter, and how do these challenges vary among different academic groups?

RQ3: What strategies do they employ to address these challenges, and how do these strategies vary among different academic groups?

## Method

To address the research questions outlined above, we adopted a mixed-methods approach, integrating quantitative data collection through a questionnaire survey with qualitative insights obtained from semi-structured interviews.

### Ethics statement

This study, entitled “Linguistic Challenges of Writing Papers in English for Scholarly Publication: Perceptions of Chinese Academics in Science and Engineering,” was conducted in strict adherence to ethical guidelines for research involving human participants.

#### Ethical approval.

The research protocol was reviewed and approved by the Ethics Committee of Graduate School of International Cultural Studies, Tohoku University, with the approval number: 2023-13. The committee's approval confirms that the study meets the ethical standards required for research involving human participants. The original approval document from the Ethics Committee is uploaded.

#### Inclusivity in global research.

Additional information regarding the ethical, cultural, and scientific considerations specific to inclusivity in global research is included in the (S1 File).

#### Participant recruitment.

The recruitment of participants for this study involved distributing questionnaires and conducting semi-structured interviews. The recruitment period commenced on 23th, September, 2023 and concluded on 30th, November, 2023. Participants included university teachers, researchers, doctoral and master's students.

#### Informed consent.

All participants were fully informed about the nature and purpose of the study. Written informed consent was obtained from each participant prior to their involvement in the study. Participants in the questionnaire survey must check the option agreeing to participate in the survey before they can proceed with the questionnaire. Therefore, all the valid questionnaires received are from participants who have given their consent. All thirteen interviewees provided signed consent to participate.

The consent provided participants with detailed information about the study's objectives, procedures, potential risks, and benefits. Participants were assured of the confidentiality and anonymity of their responses. They were informed that participation was voluntary and that they could withdraw from the study at any time without any negative consequences.

### Participants

#### Questionnaire respondents.

The target respondents for the questionnaire were Chinese academics in the fields of science and engineering, including university teachers, institute researchers, doctoral students, and master’s students. We employed purposive sampling to recruit participants who met the following three criteria: (1) being a Chinese native of mainland China; (2) being at least a master-level student or higher, or currently employed as a university teacher or institute researcher (undergraduate students were excluded due to their limited experience with thesis or paper writing, which could lead to speculative responses); (3) specializing in science or engineering. The questionnaire link was distributed via accessible online platforms to targeted groups of Chinese academics, with participation being entirely voluntary.

Out of 856 questionnaires received, we identified 732 as valid for analysis, resulting in an 86% response rate. The respondents comprised 87 university teachers or researchers, 119 doctoral students, and 526 master’s students, all specializing in science or engineering discipline (Table 1). These respondents were drawn from a range of provinces across mainland China.

**Table 1:** Composition of questionnaire respondents.

university teachers/researchers	87
doctoral students	119
master's students	526
Total	732

#### Interview participants.

To ensure the diversity and representativeness of the sample, semi-structured interviews were conducted with 13 participants from various academic roles, ranks and disciplines, selected through purposive sampling [40]. This method allows for targeted selection of individuals with specific knowledge relevant to the research questions. The sample included 1 associate professor in foreign languages and applied linguistics (who taught English academic writing), 2 professors, 2 researchers, 1 post-doctoral researcher, 4 doctoral students, and 2 master's students from different scientific and engineering disciplines. All participants were selected based on their experience with publishing papers in English and their willingness, thereby ensuring that their insights would directly align with the objectives of the research. Efforts were made to include a mix of career stages to capture a comprehensive range of perspectives, which is essential for a robust qualitative analysis [41]. To protect participants’ privacy and confidentiality, pseudonyms were used throughout the study. This approach is consistent with established qualitative research practices aimed at maximizing diversity and representativeness while upholding ethical standards. The demographic features are presented in Table 2.

**Table 2:** Demographic features of interviewees.

Interviewees	Identity	Disciplines	Paper published/ in process (In English)
1.Yan	Associate Professor	Foreign Languages and Applied Linguistics	10
2.Chen	Professor	Mechanical Manufacture and Automation	30
3.Meng	Professor	Materials Physics and Chemistry	20
4.Wu	Associate Researcher	Optical Communication	6
5.Zhan	Associate Researcher	Marine Science and Technology	9
6.Liu	Post-doc	Material Processing Engineering	6
7.Mi	4th Year Doctoral student	Precision Instruments and Machinery	4
8.Rong	4th Year Doctoral student	Mechanics	4
9.Zhuang	1st Year Doctoral student	Quantum Optics	2
10.Kai	2nd Year Doctoral student	Mechanical Design	1 under review
11.Fang	1st Year Doctoral student	Mechanics	1 under review
12.Chang	3rd Year master's student	Mechanical Manufacture and Automation	2 under review
13.Wen	3rd Year master's student	Mechanical Manufacture and Automation	2 under review

Of all participants, except one interviewee affiliated with the research institute, the rest are from over 50 Chinese universities across different provinces in mainland China, including Tsinghua University, Xi’an Jiaotong University, Northwestern Polytechnical University, Chang’an University, Harbin Institute of Technology, Shandong University, University of Chinese Academy of Sciences, Fudan University, Zhejiang University, Wuhan University, Shenzhen University and so on.

### Theoretical framework and procedures

This study comprised two phases. In the first phase, we designed the questionnaire (S2 File) and interview questions (S3 File). In the second phase, we conducted the questionnaire survey and interviews.

The questionnaire includes five sections with 24 items in total, in which item 5–6, 8–12, 14–17, 18,22 were designed into 6-point Likert-scale questions. Section 1 (Item 1–4) collected basic demographic information, which was essential for contextualizing the findings. Section 2 (Item 5–7) assessed the respondent' perceptions of the importance of writing in English and their experiences with publishing English-language papers. Section 3 (Item 8–13) focused on specific linguistic challenges encountered by respondents when writing in English. Section 4 (Item 14–20) explored the strategies employed to address these challenges. Section 5 (Item 21–23) examines respondent' experiences with academic writing courses and their perceptions of the benefits of such training. Item 24 invited respondents to share any additional thoughts or insights regarding the challenges and strategies of writing papers in English for scholarly publication.

While the questionnaire was designed to collect quantitative data, the interview questions aimed to elicit qualitative insights by exploring personal experiences in greater depth and capturing rich, detailed narratives that the closed-ended questionnaire format couldn't provide. For example, Q1 (Interview)--“Is writing papers in English important for your career or study? Why is it important and how does it relate to your career and study?”--builds upon the questionnaire by prompting participants to explain the significance of English academic writing in their specific contexts, thereby uncovering underlying motivations. Similarly, Q6 (Interview)--”What are the challenging aspects of writing papers in English? Why are these aspects challenging for you?”--encourages participants to elaborate on difficulties, provide concrete examples and explain the specific reasons behind their struggles. Challenge like sentence construction or coherence cannot always be fully explained through a Likert-scale response. Open-ended interview questions like Q6 invite detailed examples, making it possible to identify patterns that may not emerge from numeric data alone [40]. Thus, collecting qualitative data through interviews enhances the reliability and validity of the findings by triangulating with quantitative results [41], contributing to a more comprehensive and well-rounded understanding of the issue.

The questionnaire and interview questions designed are grounded in a theoretical framework that encompasses various dimensions of language acquisition, academic writing, and the specific challenges encountered by non-native English speakers. This framework also integrates insights from recent research on the role of technology and strategies in overcoming these challenges, and the perceptions of academic writing among non-native English-speaking scholars.

The difficulties associated with writing in English for scholarly publication are well-documented. Flowerdew [42] discusses the linguistic disadvantages that scholars face, emphasizing that these challenges extend beyond mere grammar and vocabulary to include issues of coherence and cohesion in academic writing. This is particularly relevant for Chinese academics, who may struggle with the syntactic and rhetorical conventions of English academic discourse. Similar conclusions can be drawn from Mungra and Webber [43], who found that ‘not well written’, ‘lack of clarity’ and ‘incoherent' were the most common feedback at the lexis and syntax level; and ‘text structuring to improve readability' was the common concern on the level of discourse. Furthermore, Guo, Hamat, and Jaafar [44] conducted an extensive review of the literature on L2 writing anxiety, highlighting the challenges faced by non-native writers as a result of additional physiological and psychological stressors.

Kashiha [45] analyzed the use of cohesive markers by ESL learners and found that many students appeared uncertain about the significance of employing cohesive devices in their essays. The study provided insights into common pitfalls that contribute to disjointed and incoherent writing. Similarly, Anindita [46] reviewed the literature to identify common challenges faced by non-native writers in achieving coherence and cohesion in their academic writing. She emphasized the need for targeted pedagogical interventions to support the development of these skills among non-native English-speaking students.

The questions we designed also explore the strategies that academics employ to navigate these linguistic challenges, whose design is based on previous research. For example, Bakla and Karakaş [47] illustrate how technology and various writing strategies can assist non-native speakers in improving their academic writing skills. In the research by Shamsi and Osam [48] as well as Phyo, Nikolov & Hódi [49], seeking assistance from advisors and native speakers, as well as utilizing professional editing services, are common strategies identified in the literature. These strategies are crucial for enhancing the quality of academic writing and for building confidence among non-native authors.

This framework provides a solid foundation for understanding the complexities of writing in English for scholarly publication among Chinese academics, guiding the development of the questionnaire and its subsequent analysis.

The reliability of the questionnaire is assessed using Cronbach’s alpha, with a threshold of 0.819, which suggests a high level of internal consistency among the items and affirms that the questionnaire is a reliable instrument for capturing the intended dimensions of the construct being measured. The validity of the instrument was tested with the KMO (KaiserMeyer-Olkin) value with 0.872, which suggests that the sample is adequate for factor analysis, indicating a high degree of inter-correlation among the variables. Additionally, the Bartlett’s Test of Sphericity yielded a chi-square value of 2391.634 with 190 degrees of freedom, and a *p*-value less than 0.001, confirming that the correlation matrix is not an identity matrix, thus justifying the factor analysis.

### Data collection and analysis

We distributed and collected questionnaires using the online tool Wenjuanxing (https://www.wjx.cn). The data collected were coped with SPSS 27.

When analyzing the questionnaire data using SPSS, we assigned scores to the six options of the 6-point Likert-scale as follows: *Strongly Agree* *=* *6*, *Agree* *=* *5*, *Somewhat Agree* *=* *4*, *Somewhat Disagree* *=* *3*, *Disagree* *=* *2*, and *Strongly Disagree* *=* *1*. For item Q7, respondents who had previously published articles in English were assigned a score of 2, while those who had not published were assigned a score of 1.

When analyzing the questionnaire data, we found that responses to the scale-type items exhibited non-normal distributions. To enhance the scientific rigor of the analysis, we applied Kruskal-Wallis Test and pairwise comparisons within the framework of non-parametric tests, which ranks all data points across groups and compares the ranks. As Ostertagová et al. [50] note, the Kruskal-Wallis test is advantageous in situations where the underlying population distributions are unknown or not normally distributed. It enables researchers to draw valid conclusions from their data without the constraints imposed by parametric tests.

We conducted all semi-structured interviews via Tencent Meeting. Except for six students who participated in individual interviews averaging forty minutes, the remaining interviewees took part in group interviews lasting nearly two hours. Conducted in Chinese, all interviews were recorded for subsequent transcription and analysis. We analyzed the interview data by identifying and summarizing recurring themes, ensuring a thorough understanding of the responses.

## Results and discussion

**Question 1:** What are the attitudes of Chinese academics toward writing papers in English for scholarly publication, and what is their current experience with publishing in English-language journals?

### Attitudes toward writing papers in English for scholarly publication

We calculated the percentages of the respondents in each academic group who chose “strongly agree” and “agree”. 97.70% of university teachers and researchers, 94.96% of doctoral students, and 79.47% of master's students agreed that “English paper publication is very important for your career or academic pursuit”, which suggests that academics recognize the direct impact of such publications on their professional growth and visibility in the global academic community. To examine whether there were differences among the three academic groups regarding the attitude towards publication, we applied Kruskal-Wallis Test (Table 3). The results indicated no statistically significant differences among the groups (Asymp. Sig. = .538, *p *> .05).

**Table 3:** Kruskal-Wallis test for comparing the attitudes toward paper publication in English.

Questionnaire Q5	Groups	N	Mean rank	Kruskal-Wallis H	df	Asymp. Sig.
Writing papers in English is important.	1	87	378.34	1.239	2	.538
	2	119	375.41			
	3	526	362.53			
	Total	732				

1. university teachers and researchers.

2. doctoral students.

3. master's students.

The responses from the interviewees align with the results of the questionnaire, confirming the critical role of English-language paper publication in advancing both academic and career prospects. One doctoral student noted:

*“Our university requires us to publish at least two journal papers, including one in an SCI Q2-level journal, in order to qualify for a doctoral degree.” (Interviewee 8: Doctoral student Rong)*

Another interviewee emphasized the importance of quality alongside quantity:

*“The emphasis is not only on the number of publications but also on their quality. Publishing in high-impact journals can lessen the required number of publications.” (Interviewee 7: Doctoral student Mi)*

A postdoctoral researcher further highlighted the impact of high-quality publications on career advancement:

*“I graduated with six first-authored SCI papers as a doctoral candidate. High-quality publications were instrumental in securing my prestigious academic position.” (Interviewee 6: Postdoctoral researcher Liu)*

These responses are consistent with the findings of Shamsi and Osam [48], who identified a “publish or no degree” environment for non-native English-speaking doctoral students. Moreover, previous research by Khoo, Zegwaard, & Adam [51] has underscored that the ability to effectively communicate research findings in English is a crucial skill for these students, directly influencing both their academic success and future career opportunities.

Furthermore, doctoral student Wen and master's student Zhuang acknowledged the significance of publishing in SCI or EI journals for securing scholarship opportunities and gaining admission to doctoral programs. This observation aligns with previous studies, which have shown that academic publications in high-impact journals are often pivotal for students’ academic advancement. For instance, A strong publication history enhances the academic credentials of graduate students, making them more competitive in the employment market and supporting successful efforts, including obtaining research grants and pursuing advanced academic opportunities [52,53]. Additionally, Khoo et al. [51] highlighted that such publications are increasingly regarded as essential for demonstrating academic competence and distinguishing oneself in the competitive academic and professional job market.

The publication requirements impose considerable pressure on both graduate students and university faculty. Interviewee Professor Chen and researcher Wu discussed the “no-promotion means departure” policy at certain institutions, where failure to publish in SCI journals could jeopardize academic positions, underscoring the intense pressure associated with tenure-track contracts that require meeting publication criteria.

As Professor Chen explained:

*“Look at my gray hair; you might be surprised to learn that I’m only in my thirties. Besides my teaching duties, I have to publish at least four papers during my first four-year tenure-track contract.” (Interviewee 2: Professor Chen)*

Similarly, Researcher Wu reflected on the widespread expectation across academic institutions:

*“From my job-hunting experiences, nearly all universities and institutions require publications, which are essential for retaining a position or securing promotion.” (Interviewee 4: Researcher Wu)*

In the context of China, Ge [54] observed a growing emphasis on publishing in English-language journals, driven by national research policies and a desire to participate in the international academic community. This push to publish in high-impact, English-language journals, as Chen, Ren, Yang & Abudouguli noted [55], is particularly pronounced in the natural sciences and engineering disciplines, where Chinese scholars are actively engaging with the global research community.

### Attitudes toward importance of English language in scholarly publication

Similar to Q5, we calculated the percentages of the respondents in each academic group who chose “strongly agree” and “agree”. We found 87.36% of university teachers and researchers, 90.11% of doctoral students, and 84.32% of master's students acknowledged the critical role of English language in scholarly publication. The *p*-value (Asymp. Sig. = .170, *p *> 0.05) in Table 4 indicates no significant difference across these groups, suggesting a shared recognition of the importance of language in the academic publishing process. This consensus aligns with previous research that emphasizes the centrality of language proficiency in facilitating effective communication of scientific ideas.

**Table 4:** Kruskal-Wallis test for attitudes toward importance of English language in scholarly publication.

Questionnaire Q6	Groups	N	Mean rank	Kruskal-Wallis H	df	Asymp. Sig.
English is important for paper writing.	1	87	340.07	3.546	2	.170
	2	119	351.11			
	3	526	374.35			
	Total	732				

^1^. university teachers and researchers 2. doctoral students 3. master's students.

The interview data suggest that language-related issues frequently lead to manuscript rejection, with editors and reviewers often highlighting language problems or advising authors to seek professional language editing services. This echoes the research by Datta and Jones [56], who identified insufficient English proficiency as a primary reason for manuscript rejection, supporting earlier studies that language-related issues are significant barriers to publication [42,47,57]. Some researchers, including Hanauer & Englander [19], Politzer-Ahles et al. [58] and Li [59], found that non-native English-speaking authors often face greater challenges writing research articles in English than native English counterparts, a difficulty particularly pronounced among Chinese scholars. Moreover, Phyo et al. [60] revealed that novice NNES writers frequently enter PhD programs lacking essential English academic writing skills, presenting substantial barriers to promptly initiating doctoral-level writing tasks. These findings underscore the critical need for comprehensive support aimed at enhancing English language proficiency and providing academic writing assistance.

As one interviewee noted:

*“Articles in prestigious international journals are more enjoyable to read due to their fluent language, logical organization, and coherence.” (Interviewee 10: Doctoral student Kai)*

Beyond language issues, interviewees Mi, Rong, Liu, and Fang expressed concerns about potential disadvantages and biases compared to native English-speaking authors. This sentiment aligns with the research by Flowerdew [42], who reported that approximately one-third of the 600 Hong Kong Cantonese L1 academics felt discriminated by native English-speaking editors and referees. Similarly, Politzer-Ahles et al. [61] provided evidence of linguistic bias in academic peer-review processes, further indicating that non-native English-speaking authors often face additional hurdles in publication.

However, Professor Chen, a reviewer for multiple international journals, emphasized that reviewers do not reject papers solely based on the author's identity or their non-native language proficiency. His perspective is consistent with Hyland [16], who observed the growing presence of scholars using English as an Additional Language (EAL) scholars within global scholarly communication. In a related study with Habibie [62], Hyland further concluded that EAL authors do not face language-based injustices when publishing in English. They also pointed out that academic publishing challenges are faced by scholars worldwide, irrespective of nationality and therefore it is unfair to blame reviewers and editors for prejudice. Reviewers were generally reported to provide constructive feedback, while editors were seen as making fair and reasonable decisions based on the scholarly quality of the submissions. Similarly, Campos-Arceiz et al. [14] found no evidence of editorial bias against papers from China compared to those from English-speaking countries.

These contrasting perspectives highlight the complexity of the issue. While perceptions of linguistic and cultural bias persist, empirical evidence suggests that academic publishing challenges are broadly shared among scholars worldwide, regardless of nationality. Given these nuances, further research incorporating a broader dataset and interviews is needed to deepen understanding of these dynamics and to provide a more comprehensive analysis of perceived and actual biases in international academic publishing.

### English paper publication experiences

Table 5 presents the English-language publication rates across different academic groups. Specifically, 83.91% of university teachers and researchers have published papers in English, compared to 63.03% of doctoral students and only 14.45% of master's students. The *p*-value (Asymp. Sig. < .001) indicates a significant disparity among the three groups. The engagement in English-language scholarly publication increases notably with academic seniority. The higher publication rate among university teachers and researchers likely reflects their greater experience, broader access to academic resources, and institutional expectations to publish in English to enhance their professional standing and contribute to global academic discourse. Similarly, doctoral students tend to have more extensive research experience, have completed more coursework and engaged in independent research projects compared to master’s students. This prior exposure to research and academic writing equips doctoral students with the skills and knowledge necessary for more effective engagement in publishing activities. In contrast, the lower publication rates among master’s students may reflect their limited experience and the challenges they face in producing research of publishable quality in a non-native language. This trend highlights the progressive development of academic proficiency and underscores the critical role of English in scholarly communication at advanced stages of an academic career.

**Table 5:** Kruskal-Wallis Test for English paper publication experiences.

Questionnaire Q7	Groups	N	Mean rank	Kruskal-Wallis H	df	Asymp. Sig.
Have you ever published papers in English?	1	87	561.60	239.615	2	<.001
	2	119	485.17			
	3	526	307.38			
	Total	732				

^1^. university teachers and researchers 2. doctoral students 3. master's students.

Question **2:** What linguistic challenges do they encounter, and how do these challenges vary among different academic groups?

### Results from questionnaire data

Table 6 summarizes the mean ranks for various aspects of writing challenges across different academic groups, while Tables 7–9 presents pairwise comparisons to identify the most and least challenging aspects in each academic group, including their statistical significance.

**Table 6:** Mean ranks of linguistic challenges in academic groups.

university teachers and researchers			doctoral students		master's students	
Challenges	N	Mean Rank	N	Mean Rank	N	Mean Rank
grammar	87	124.67	119	213.87	526	1239.53
words	87	244.91	119	343.71	526	1321.56
sentence construction	87	279.22	119	361.61	526	1444.77
cohesive devices	87	253.12	119	323.75	526	1271.33
coherence	87	188.07	119	247.06	526	1300.30

**Table 7:** Pairwise comparisons of challenges for university teachers and researchers.

	Test Statistic	Std. Error	Std. Test Statistic	Sig.	Adj. Sig.^a^
1-5(grammar-coherence)	-63.408	18.180	-3.488	.000	.005
1-2(grammar-words)	-120.247	18.180	-6.614	.000	.000
1-4(grammar-cohesive devices)	-128.454	18.180	-7.066	.000	.000
1-3(grammar- sentence construction)	-154.557	18.180	-8.502	.000	.000
5-2(coherence-words)	56.839	18.180	3.127	.002	.018
5-4(coherence-cohesive devices)	65.046	18.180	3.578	.000	.003
5-3(coherence-sentence construction)	91.149	18.180	5.014	.000	.000
2-4(words-cohesive devices)	-8.207	18.180	-.451	.652	1.000
2-3 (words-sentence construction)	-34.310	18.180	-1.887	.059	.591
4-3(cohesive devices-sentence construction)	26.103	18.180	1.436	.151	1.000

The significance level is.050.

a. Significance values have been adjusted by the Bonferroni correction for multiple tests.

**Table 8:** Pairwise comparisons of challenges for doctoral students.

	Test Statistic	Std. Error	Std. Test Statistic	Sig.	Adj. Sig.^a^
1-5(grammar-coherence)	-33.197	20.916	-1.587	.112	1.000
1-4(grammar-cohesive devices)	-109.882	20.916	-5.254	.000	.000
1-2(grammar-words)	-129.845	20.916	-6.208	.000	.000
1-3(grammar-sentence construction)	-147.748	20.916	-7.064	.000	.000
5-4(coherence-cohesive devices)	76.685	20.916	3.666	.000	.002
5-2(coherence-words)	96.647	20.916	4.621	.000	.000
5-3(coherence-sentence construction)	114.550	20.916	5.477	.000	.000
4-2(cohesive devices-words)	19.962	20.916	.954	.340	1.000
4-3(cohesive devices-sentence construction)	37.866	20.916	1.810	.070	.702
2-3(words-sentence construction)	-17.903	20.916	-.856	.392	1.000

The significance level is.050.

a. Significance values have been adjusted by the Bonferroni correction for multiple tests.

**Table 9:** Pairwise comparisons of challenges for master’s students.

	Test Statistic	Std. Error	Std. Test Statistic	Sig.	Adj. Sig.^a^
1-4(grammar-cohesive devices)	-31.807	41.625	-.764	.445	1.000
1-5(grammar-coherence)	-60.778	41.625	-1.460	.144	1.000
1-2(grammar-words)	-82.038	41.625	-1.971	.049	.487
1-3(grammar- sentence construction)	-205.244	41.625	-4.931	.000	.000
4-5(cohesive devices-coherence)	-28.971	41.625	-.696	.486	1.000
4-2(cohesive devices-words)	50.231	41.625	1.207	.228	1.000
4-3(cohesive devices-sentence construction)	173.437	41.625	4.167	.000	.000
5-2(coherence-words)	21.260	41.625	.511	.610	1.000
5-3(coherence-sentence construction)	144.467	41.625	3.471	.001	.005
2-3(words-sentence construction)	-123.206	41.625	-2.960	.003	.031

The significance level is.050.

a. Significance values have been adjusted by the Bonferroni correction for multiple tests.

For university teachers and researchers, constructing proper sentences (Mean Rank = 279.22), using appropriate words (Mean Rank = 244.91) and cohesive devices (Mean Rank = 253.12) are three primary challenges with no statistical difference between them: words vs. sentences (T = -1.887, Adj. Sig = .591), words vs. cohesive devices (T = -8.207, Adj. Sig = 1.000); cohesive devices vs. sentences (T = 26.103, Adj. Sig = 1.000). Coherence (Mean Rank = 188.07) ranks below these aspects with statistical difference: coherence vs. words (T = 56.839, Adj. Sig = .018), coherence vs. sentence construction (T = 91.149, Adj. Sig = .000), coherence vs. cohesive devices (T = 65.046, Adj. Sig = .003). Grammar (Mean Rank = 124.67) is the least challenging aspect overall, significantly less regarded as a challenge than other aspects of linguistic components: grammar vs. words (T = -120.247, Adj. Sig = .000), grammar vs. sentence construction (T = -154.557, Adj. Sig = .000), grammar vs. cohesive devices (T = -128.454, Adj. Sig = .003), grammar vs. coherence (T = -63.408, Adj. Sig = .005).

Doctoral students reported similar patterns. Sentence construction (Mean Rank = 361.61), appropriate word choice (Mean Rank = 343.71) and the use of cohesive devices (Mean Rank = 323.75) were cited as major hurdles, yet no significant differences were detected among them: words vs. sentence construction (T = -17.903, Adj. Sig = 1.000), cohesive devices vs. words (T = 19.962, Adj. Sig = 1.000), cohesive devices vs. sentence construction (T = 37.866, Adj. Sig = 1.000). Coherence (Mean Rank = 188.07) followed with statistically significant distinctions when compared to words (T = 96.647, Adj. Sig = .000), sentence construction (T = 114.550, Adj. Sig = .018), and cohesive devices (T = 76.685, Adj. Sig = .003). Grammar (Mean Rank = 124.67) again emerged as the least challenging, with significant differences from other aspects: grammar vs. words (T = -129.845, Adj. Sig = .000), grammar vs. sentence construction (T = -147.748, Adj. Sig = .000), grammar vs. cohesive devices (T = -109.882, Adj. Sig = .002), though it did not differ significantly from coherence (T = -33.197, Adj. Sig = 1.000).

For master's students, sentence construction was identified the most significant challenge (Mean Rank = 361.61), showing no statistically significant differences when compared to grammar (T = -205.244, Adj. Sig = .000), words (T = -123.206, Adj. Sig = .031), cohesive devices (T = 173.437, Adj. Sig = .000), and coherence (T = 144.467, Adj. Sig = .005). In contrast, the remaining four aspects--words, cohesive devices, coherence, and grammar--were perceived similarly difficult, with no statistically differences observed among them.

The results indicate a clear pattern across all academic groups, with constructing appropriate sentences consistently identified as the most challenging aspect and grammar as the least challenging aspect of English academic writing across all groups. Interestingly, the lack of statistically significant differences among words, cohesive devices, and sentence construction for both university teachers and researchers as well as doctoral students suggests that these challenges are perceived at similar difficulty levels across these groups. This finding indicates a shared experience among more advanced academics regarding the complexities of English academic writing.

As shown in Tables 10 and 11, master's students perceived grammar and coherence as significantly greater challenges compared to both doctoral students and university teachers and researchers. Regarding grammar, the difference between Group 1 (university teachers and researchers) and Group 3 (master's students) was significant, with a test statistic of -200.928 and an adjusted significance of 0.000. Similarly, the comparison between Group 2 (doctoral students) and Group 3 also revealed statistical significance, with a test statistic of -135.050 and an adjusted significance of 0.000. 

**Table 10:** Pairwise comparisons of grammar as challenge in academic groups.

	Test Statistic	Std. Error	Std. Test Statistic	Sig.	Adj. Sig.^a^
1-2	-65.878	28.492	-2.312	.021	.062
1-3	-200.928	23.378	-8.595	.000	.000
2-3	-135.050	20.504	-6.587	.000	.000

Asymptotic significances (2-sided tests) are displayed. The significance level is.050.

a. Significance values have been adjusted by the Bonferroni correction for multiple tests.

1. university teachers and researchers 2. doctoral students 3. master's students

**Table 11:** Pairwise comparisons of coherence as challenge in academic groups.

	Test Statistic	Std. Error	Std. Test Statistic	Sig.	Adj. Sig.^a^
1-2	-5.170	27.547	-.188	.851	1.000
1-3	-113.848	22.603	-5.037	.000	.000
2-3	-108.678	19.824	-5.482	.000	.000

Asymptotic significances (2-sided tests) are displayed. The significance level is.050.

a.Significance values have been adjusted by the Bonferroni correction for multiple tests.

1. university teachers and researchers 2. doctoral students 3. master's students

For coherence, significant differences were also observed. The comparison between Group 1 (university teachers and researchers) and Group 3 (master's students) yielded a test statistic of -113.848 and an adjusted significance of 0.000. Likewise, Group 2 (doctoral students) and Group 3 showed a significant difference, with a test statistic of -108.678 and an adjusted significance of 0.000.

### Findings from interviews and discussion

#### Using correct grammar.

During the interviews, only one doctoral student, Zhuang, mentioned grammar-related issues; However, he did not perceive them as a significant challenge. He attributed occasional grammatical lapses to a lack of recent formal study but noted that he addressed these issues by consulting grammatical rules as needed.

Non-native English speakers often perceive grammar as a significant barrier to academic publication due to lower confidence in their English proficiency. Politzer-Ahles et al. [61] reported that non-native speakers frequently doubt their grammatical accuracy, which can divert attention from the substantive content of their writing. Similarly, Hyland [63] noted that early English education for non-native speakers often overemphasizes grammatical accuracy at the expense of higher-order writing skills such as coherence, organization, and argumentation. This emphasis may lead to a perception of grammar as a more significant barrier than other aspects of academic writing, such as sentence construction or overall coherence.

Master’s students, in particular, are more likely to perceive grammar as a linguistic challenge, owing to limited academic writing experience, insufficient training, and the residual effects of grammar-focused instruction. Unlike doctoral programs--which often incorporate academic writing development through coursework, research activities, and publication requirements--master's programs typically prioritize coursework over preparation for scholarly publication [64]. This lack of exposure to academic writing conventions can leave many master's students underprepared for the grammatical demands of academic journals. Furthermore, limited experience with academic publishing process may intensify this perception, making grammar appear as a more noticeable challenge. The unfamiliarity with the peer-reviewed publication protocols likely exacerbates the sense that grammatical proficiency is a critical shortcoming.

#### Using proper words.

Two key vocabulary-related challenges emerged from the interview data. First, selecting appropriate words in academic contexts posed significant difficulties, resulting in unclear expressions and repetitive language. This finding aligns with Sun’s survey [65], in which second-year doctoral students identified English expression and word choice as major obstacles in academic writing. Second, participants reported struggles with handling complex noun strings, a difficulty particularly pronounced in scientific and engineering disciplines. As Mi, a doctoral student, explained:

*“Technical terms in science and engineering are often very long, and I struggle with how to handle these multi-word terms, especially when writing paper titles. Combining these terms concisely while retaining their original meaning is particularly challenging.”*

Mi’s concerns reflect findings of Ndoricimpa [66], which highlight the complexity of employing discipline-specific terminology in scholarly writing. Such challenges often lead to miscommunication or ambiguity, especially in scientific contexts where precision and clarity are critical.

The interviewees identified several underlying causes for these issues, including limited academic vocabulary, unfamiliarity with nuanced word usage, and negative transfer from their native language. For instance, Rong, a doctoral student, noted:

*“As a non-native English speaker, I often think in Chinese and choose words that appear similar but are inappropriate for academic writing. My limited vocabulary and lack of formal training in academic writing also contribute to repetitive language and imprecise word choices, which may confuse readers.”*

These findings are consistent with Hyland [63], who demonstrated that poor vocabulary often results in imprecise or contextually inappropriate word choices. The linguistic disparity between Chinese--a logographic language--and English--an alphabetic language, further compounds these challenges. Prior research [67,68] indicates that this structural difference complicates word recognition and selection in academic English for Chinese authors, resulting in ambiguity and miscommunication.

The pressure to publish in high-impact English-language journals further intensifies these challenges. Non-native authors, acutely aware of their lexical limitations, often experience anxiety and self-doubt [69]. This heightened pressure can undermine confidence in vocabulary selection, rendering the writing process more daunting and less effective.

#### Constructing appropriate sentences.

Sentence construction emerged as a pervasive challenge across all academic groups. The questionnaire results were consistent with interview data, revealing widespread difficulties in balancing short and long sentences and navigating syntactic structures. For example, Zhan, a researcher, explained:

*“When writing papers, I always rack my brains to craft complex sentences because I believe long sentences make me appear more knowledgeable. However, I also read in the APA guidelines that scientific papers should avoid overly long sentences and be concise and clear. At the same time, it mentions that having too many short sentences can make the writing choppy. Therefore, I am quite confused about how to balance long and short sentences effectively.” (Interviewee 5: Researcher Zhan)*

Similarly, Doctoral Student Zhuang remarked:

*“I try my best to express my ideas clearly. However,* reviewers *have pointed out that my sentences are convoluted and hard to understand. When I revise, I can’t find any grammatical or structural errors, so I feel completely at a loss.” (Interviewee 9: Doctoral Student Zhuang)*

These challenges stem from fundamental linguistic differences between Chinese and English. English syntactic structures and grammatical rules often conflict with the native patterns in Chinese, resulting in awkward phrasing and inconsistencies [70]. In addition, non-native English-speaking authors must navigate the additional complexity academic writing conventions, which vary across disciplines and impose distinct expectations for sentence complexity and clarity. Politzer-Ahles et al. [61] noted that language bias in academic reviews can exacerbate these difficulties, as reviewers may apply stricter standards to non-native authors, thereby intensifying the pressure to conform to normative expectations. The high stakes of publishing in competitive, high-impact journals further magnify these challenges. Authors must meet stringent publication standards, which often leads to heightened anxiety and self-doubt, complicating their ability to craft clear and effective sentences [71].

#### Using proper cohesive devices.

Interviewees mentioned incorrect use or overuse of cohesive devices. Rong, a fourth-year doctoral student, reported paying close attention to cohesion between sentences but acknowledged misunderstanding regarding the appropriate use of cohesive markers. For example, she noted an overuse of connectives such as “*but*,” “*however*,” and various adverbs indicating sequence. While such connectives may superficially signal logical relationships, their excessive use can undermine sentence-level logic and disrupt the overall coherence of the text.

Some scholars have observed that Chinese authors often rely heavily on summary transitions, reflecting problematic use of cohesive devices [72–74]. Over-reliance on these markers can produce a disjointed or artificial flow of ideas, ultimately reducing overall coherence and readability.

Flowerdew [42] further indicated that many EFL (English as a Foreign Language) learners struggle to employ cohesive devices effectively, frequently resulting in fragmented and disjointed prose that impedes the reader's ability to follow the argument. Therefore, while cohesive devices play a critical role in establishing textual coherence, their incorrect or excessive use may diminish the overall quality and clarity of academic writing.

#### Organizing content with coherence.

Maintaining coherence in academic writing was identified as both essential and challenging. Interviewees emphasized that while individual sentences were often grammatically correct, their overall flow was frequently disjointed. Yan, an academic writing instructor, explained:

*“I have often observed that students first write papers in Chinese and then translate them into English, which inevitably carries over Chinese thinking patterns. English thinking is straightforward, while Chinese thinking tends to be more circuitous. As a result, what is easily understood in Chinese becomes confusing in English, not conforming to the conventions of English academic writing.” (Interviewee 1: Academic English writing instructor Yan)*

This negative transfer of Chinese rhetorical conventions into English writing often results in fragmented coherence, highlighting the importance of mastering cohesion. As Halliday and Hasan [75] emphasize, effective cohesion is fundamental to producing coherent texts. The research by Tso [76] also showed that Chinese EFL learners often struggle to produce fluent, cohesive English texts with clear logical flow. This challenge is largely attributed to the profound cultural and rhetorical differences between Chinese and English, even among learners with a solid understanding of English grammar and vocabulary.

#### Summary.

The challenges outlined above are interrelated, with difficulties in one area often exacerbating those in others. For example, vocabulary choice influences grammatical collocation, which, in turn, affects sentence structure and overall coherence. This creates a cascading effect, where minor issues can permeate and undermine the entire writing process.

Several interrelated factors contribute to these challenges. Limited proficiency in English is a key barrier, as it impedes the ability to select accurate vocabulary, construct coherent sentences, and utilize cohesive devices, thereby hindering effective communication of ideas [3,77]. Additionally, Chinese rhetorical conventions often influence sentence construction, coherence, and grammar in English academic writing, leading to further complications [76,78,79].

The Chinese educational system also plays a significant role in these challenges. The curriculum's focus on high-stakes standardized testing and the restricted language environment within universities often result in limited development of writing skills and strategies for English academic contexts [80]. Furthermore, the absence of formal training in academic writing conventions leaves many Chinese scholars ill-prepared for the demands of international publication [81].

Language transfer and related misconceptions represent additional obstacles. The transfer of first-language (L1) writing conventions into second-language (L2) academic texts, coupled with misconceptions regarding proper language use, can negatively impact sentence structure and coherence [48,82].

**Question 3:** What strategies do they employ to address these challenges, and how do these strategies vary among different academic groups?

### Results from questionnaire data

A total of 411 participants (80 university teachers and researchers, 97 doctoral students, and 234 master's students) responded to this question in the questionnaire. Table 12 summarizes the mean ranks for various strategies across different academic groups, while Tables 13–15 present pairwise comparisons to identify the most and least used strategies for each group.

**Table 12:** Mean rank of strategies in academic groups.

University Teachers and Researchers			Doctoral students		master's students	
Strategies	N	Mean Rank	N	Mean Rank	N	Mean Rank
1 Online platforms and software tools	80	259.99	97	313.98	234	835.38
2 Assistance from advisors	80	83.20	97	283.09	234	641.72
3 Assistance from native Chinese speakers	80	264.81	97	296.06	234	754.84
4 Assistance from native English speakers	80	177.47	97	97.50	234	275.59
5 Editing companies	80	217.03	97	224.38	234	419.97

**Table 13:** Pairwise Comparisons of strategies by university teachers and researchers.

Strategies	Test Statistic	Std. Error	Std. Test Statistic	Sig.	Adj. Sig.^a^
2-4 (AA--ANES)	-94.269	17.965	-5.247	.000	.000
2-5 (AA--EC)	-133.825	17.965	-7.449	.000	.000
2-1 (AA--OPST)	-176.794	17.965	-9.841	.000	.000
2-3 (AA--ANCS)	-181.612	17.965	-10.109	.000	.000
4-5 (ANES--EC)	-39.556	17.965	-2.202	.028	.277
4-1(ANES--OPST)	-82.525	17.965	-4.594	.000	.000
4-3 (ANES--ANCS)	-87.344	17.965	-4.862	.000	.000
5-1(EC--OPST)	-42.969	17.965	-2.392	.017	.168
5-3 (EC--ANCS)	-47.788	17.965	-2.660	.008	.078
1-3 (OPST--ANCS)	-4.819	17.965	-.268	.789	1.000

1. OPST--Online platforms and software tools.

2. AA--Assistance from advisors.

3. ANCS--Assistance from native Chinese speakers.

4. ANES--Assistance from native English speakers.

5. EC--Editing companies.

**Table 14:** Pairwise comparisons of strategies by doctoral students.

	Test Statistic	Std. Error	Std. Test Statistic	Sig.	Adj. Sig.^a^
4-5 (ANES--EC)	-126.876	19.644	-6.459	.000	.000
4-2 (ANES--AA)	185.588	19.644	9.447	.000	.000
4-3 (ANES--ANCS)	-198.557	19.644	-10.108	.000	.000
4-1 (ANES--OPST)	-216.479	19.644	-11.020	.000	.000
5-2 (EC--AA)	-58.711	19.644	-2.989	.003	.028
5-3 (EC--ANCS)	-71.680	19.644	-3.649	.000	.003
5-1(EC--OPST)	-89.603	19.644	-4.561	.000	.000
2-3 (AA--ANCS)	-12.969	19.644	-.660	.509	1.000
2-1 (AA--OPST)	-30.892	19.644	-1.573	.116	1.000
3-1(ANCS--OPST)	-17.923	19.644	-.912	.362	1.000

1. OPST--Online platforms and software tools.

2. AA--Assistance from Advisors.

3. ANCS--Assistance from native Chinese speakers.

4. ANES--Assistance from native English speakers.

5. EC--Editing companies.

**Table 15:** Pairwise comparisons of strategies by master’s students.

	Test Statistic	Std. Error	Std. Test Statistic	Sig.	Adj. Sig.^a^
4-5 (ANES--EC)	-144.385	30.406	-4.749	.000	.000
4-2 (ANES--AA)	-366.130	30.406	-12.042	.000	.000
4-3 (ANES--ANCS)	-479.259	30.406	-15.762	.000	.000
4-1 (ANES--OPST)	-559.799	30.406	-18.411	.000	.000
5-2 (EC--AA)	-221.746	30.406	-7.293	.000	.000
5-3 (EC--ANCS)	-334.874	30.406	-11.014	.000	.000
5-1(EC--OPST)	-415.415	30.406	-13.662	.000	.000
2-3 (AA--ANCS)	-113.128	30.406	-3.721	.000	.002
2-1 (AA--OPST)	-193.669	30.406	-6.369	.000	.000
3-1(ANCS--OPST)	-80.541	30.406	-2.649	.008	.081

For university teachers and researchers, the three most commonly selected strategies for academic writing support were assistance from native Chinese speakers (ANCS) (Mean Rank = 264.81), online platforms and software tools (OPST) (Mean Rank = 259.99), and editing companies (EC) (Mean Rank = 217.03). These strategies showed no statistically significant differences. Assistance from native English speakers (ANES) followed, with statistically significant differences observed when compared to online platforms and software tools (OPST) (T = -82.525, Adj. Sig. = .000) and assistance from native Chinese speakers (ANCS) (T = -87.344, Adj. Sig. = .000), but no significant difference was found when compared to editing companies (EC). Assistance from advisors (AA) had the lowest mean rank (Mean Rank = 83.20) and exhibited significant statistical differences when compared to all other strategies, including AA vs. ANES (T = -94.269, Adj. Sig. = .000), AA vs. EC (T = -133.825, Adj. Sig. = .000), AA vs. OPST (T = 176.794, Adj. Sig. = .000), and AA vs. ANCS (T = -181.612, Adj. Sig. = .000).

Doctoral students showed a strong preference for online platforms and software tools (OPST) (Mean Rank = 313.98), assistance from native Chinese speakers (ANCS) (Mean Rank = 296.06), and assistance from advisors (AA) (Mean Rank = 283.09), with no statistical differences among them. Editing companies (EC) (Mean Rank = 224.38) ranked next in preference, while assistance from native English speakers (ANES) (Mean Rank = 97.50) was the least favored strategy. Both of them exhibited significant differences when compared to other strategies.

Among master's students, online platforms and software tools (OPST) (Mean Rank = 835.38) and assistance from native Chinese speakers (ANCS) (Mean Rank = 754.84) were the most preferred strategies, with no statistical difference between them. Assistance from advisors (AA) (Mean Rank = 641.72) ranked next. Followed by editing companies (EC) (Mean Rank = 419.97). Assistance from native English speakers (ANES) (Mean Rank = 275.59) were less favored, rated significantly lower than other strategies, including editing companies (EC) (T = -144.385, Adj. Sig. = .000), assistance from advisors (AA) (T = -366.130, Adj. Sig. = .000), assistance from native Chinese speakers (ANCS) (T = -479.259, Adj. Sig. = .000), and online platforms and software tools (OPST) (T = -559.799, Adj. Sig. = .000). Editing companies (EC) were rated lower than ANCS (T = -334.874, Adj. Sig. = .000) and OPST (Test Statistic = -415.415, Adj. Sig. = .000) as well as assistance from advisors (AA) (T = -221.746, Adj. Sig. = .000). Assistance from advisors (AA) was rated significantly lower than both OPST (T = -193.669, Adj. Sig. = .000) and ANCS (Test Statistic = -113.128, Adj. Sig. = .002).

Table 16 shows online platforms and software tools (OPST) receive considerable preference across all groups, especially among master's students with a significantly greater preference compared to university teachers and researchers (T = -41.00, Adj. Sig. = .008). Table 17 demonstrates assistance from advisors is more preferred in both doctoral and master's students, with significant difference with university teachers and researchers: Group 1 vs. Group 2 (T = -173.422, Adj. Sig. = 0.000); Group 1 vs. Group 3 (T = -144.095, Adj. Sig. = 0.000). Table 18 indicates assistance from native English speakers were more perceived as a strategy by university teachers and researchers than both doctoral students and master's students: Group 1 vs. Group 2 (T = -111.823, Adj. Sig. = 0.000); Group 1 vs. Group 3 (T = -127.048, Adj. Sig. = 0.000). According to Table 19, editing companies enjoy more preference in university teachers and researchers and doctoral students than master's students: Group 1 vs. Group 3 (T = -87.820, Adj. Sig. = 0.000); Group 2 vs. Group 3 (T = -74.738, Adj. Sig. = 0.000).

**Table 16:** Pairwise comparisons of using online platforms and software tool as a strategy in three academic groups.

Groups	Test Statistic	Std. Error	Std. Test Statistic	Sig.	Adj. Sig.^a^
1-2	-17.372	15.964	-1.088	.277	.830
1-3	-41.001	13.689	-2.995	.003	.008
2-3	-23.630	12.764	-1.851	.064	.192

Asymptotic significances (2-sided tests) are displayed. The significance level is.050.

a. Significance values have been adjusted by the Bonferroni correction for multiple tests.

1. university teachers and researchers 2. doctoral students 3. master's students.

**Table 17:** Pairwise comparisons of seeking assistance from advisors in three academic groups.

Groups	Test Statistic	Std. Error	Std. Test Statistic	Sig.	Adj. Sig.^a^
1-3	-144.095	15.105	-9.539	.000	.000
1-2	-173.422	17.615	-9.845	.000	.000
3-2	-29.328	14.085	-2.082	.037	.112

Asymptotic significances (2-sided tests) are displayed. The significance level is.050.

a. Significance values have been adjusted by the Bonferroni correction for multiple tests.

1. university teachers and researchers 2. doctoral students 3. master's students.

**Table 18:** Pairwise comparisons of seeking assistance from native English speakers in three academic groups.

Groups	Test Statistic	Std. Error	Std. Test Statistic	Sig.	Adj. Sig.^a^
3-2	-15.225	12.824	-1.187	.235	.705
3-1	-127.048	13.754	-9.237	.000	.000
2-1	-111.823	16.039	-6.972	.000	.000

Asymptotic significances (2-sided tests) are displayed. The significance level is.050.

a. Significance values have been adjusted by the Bonferroni correction for multiple tests.

1. university teachers and researchers 2. doctoral students 3. master's students.

**Table 19:** Pairwise comparisons of seeking assistance from editing companies in three academic groups.

Groups	Test Statistic	Std. Error	Std. Test Statistic	Sig.	Adj. Sig.^a^
3-2	-74.738	14.103	-5.300	.000	.000
3-1	-87.820	15.125	-5.806	.000	.000
2-1	-13.082	17.637	-.742	.458	1.000

Asymptotic significances (2-sided tests) are displayed. The significance level is.050.

a.Significance values have been adjusted by the Bonferroni correction for multiple tests.

1. university teachers and researchers 2. doctoral students 3. master's students.

### Findings from interviews and discussion

#### Online platforms and software tools.

The trend of employing online platforms and software tools reflects the widespread adoption of technological solutions, which offer convenient and cost-effective methods for academic work. Among the tools preferred by interviewees, Baidu Translate, Youdao Translate and Google Translate were most noted with the former two providing abundant example sentences and contextual accuracy. Doctoral student Rong and researcher Wu preferred drafting their articles in Chinese before translating them into English. Similarly, Ms. Yan observed that most of her students compose their papers in Chinese, then machine translation followed by revisions. Conversely, some interviewees prefer writing directly in English, using translation tools only when necessary. Translation tools are highly effective for pre-editing, post-editing, and partial translation [83].

Lee [83] highlighted Google Translate’s significant impact on improving English writing skills, particularly in terms of vocabulary, grammar, and expressions. Additionally, these tools are highly effective during the revision process, as supported by various scholars [84–86]. They also mentioned Ai tools such as ChatGPT, ERNIE Bot, and iFlytek, which play a role in translating and refining the language by enhancing the lexical diversity, sentence structures and coherence of academic papers. But Google Translate and chatGPT were not so widely used due to its limited accessibility in mainland China.

Interview respondents also employ online platforms for refining their articles, with Grammarly being a popular choice. Postdoctoral researcher Liu, Professor Meng, and Professor Chen use Grammarly’s free version, which corrects basic spelling, grammar, and punctuation errors, but does not address broader issues of expression and coherence. Prior research has praised Grammarly for reducing grammar errors, enhancing writing quality, and boosting authors’ confidence. However, its limitations include the need for a stable internet connection and occasional inaccuracies in suggestions. Furthermore, Grammarly has minimal impact on the overall structure and content of the article [87–90].

Tools such as grammar checkers, style guides, and writing assistants offer immediate feedback on language use, helping authors identify and correct grammatical errors, awkward phrasing, and stylistic inconsistencies. Ramírez-Castañeda [70] emphasizes that such tools can significantly improve the writing quality of non-native speakers, allowing them to produce more polished manuscripts that adhere to academic standards. This support is particularly crucial for authors who may lack confidence in their language skills, as it enables them to focus on content while ensuring grammatical accuracy.

#### Assistance from native Chinese speakers.

Given that non-native English speakers often struggle with grammatical accuracy, vocabulary selection, and overall writing structure, support from peers who have similar experiences becomes particularly valuable. For instance, Yang [91] found that Chinese EFL (English as a Foreign Language) students frequently face challenges in using non-finite clauses correctly, which can lead to awkward sentence constructions and reduced clarity in academic writing. By consulting with native Chinese speakers proficient in English, authors can gain context-specific insights into appropriate language use and receive guidance on overcoming these linguistic hurdles.

In the interviews, doctoral student Zhuang highlighted the support provided by a peer with advanced English proficiency, who assists fellow lab members with language editing. Similarly, doctoral student Kai relied on his senior lab colleague for paper revisions, both before submission and during the revision process. This strategy leverages individuals with advanced language skills who are capable of understanding both content and linguistic demands, making peer support a widely adopted practice among students. Luo & Hyland [17] also acknowledged the value of such collegial support. Alfehaid [92] also noted that the interplay between cultural identity and language proficiency can significantly influence the writing process for non-native authors. This shared cultural context can make it easier for authors to express their ideas and receive constructive feedback.For doctoral and master’s students, obtaining assistance from peers is often more accessible, and in some cases, even more convenient than seeking help from advisors.

Additionally,some Chinese academics seek assistance from non-colleague text mediators, such as English language instructors. For instance, Professor Chen, whose wife was a former college English teacher, often engaged in face-to-face consultations for paper revisions. By combining his subject-matter expertise with her language proficiency, they established an effective partnership for improving the quality of his manuscripts. This collaboration underscores the benefits of working with proficient English speakers who not only possess strong language skills but also understand the academic context of the manuscript. Luo & Hyland [36] similarly concluded that many Chinese academics seek support from English language colleagues rather than relying on professional editing services, which are often costly, of inconsistent quality, and sometimes raise ethical concerns. One particular advantage they identified is the effective communication facilitated by the shared native language between English teachers and authors.

However, not all scholars are as fortunate as Professor Chen. Many university teachers and researchers either lack access to such English language support or feel reluctant to seek repeated assistance from English teachers [36].

#### Assistance from advisors.

All student interviewees admit that they will send papers to advisors for revisions before they submit to the journals. Some admit that advisors provide invaluable support, guidance, and feedback, enhancing their writing skills, the quality of their work, and their ability to navigate the scholarly publication process successfully.

*“ When we publish articles, our advisor, who also serves as a co-author or corresponding author, must check the structure, logic, and language of the paper.” (Interviewee 11: Doctoral student Fang)*

*“My advisor oversees the writing, submission, and revision of the paper. He also receives feedback from the reviewers and provides me with suggestions based on their comments. Before I submit the revised manuscript, he reviews it again. From my personal perspective, I rely on my advisor for his guidance.” (Intrtviewee 12: master's student Chang)*

The advantages from this strategy are obvious and consistent with previous scholars. For example, advisor' support help students form more coherent and well-structured drafts [93,94]. Advisor-student collaboration helps students develop their writing skills, learn effective revision strategies, and communicate complex ideas clearly, boosting their confidence and competence in in academic writing [95].

However, there are different voices during interviews. Some students admitted that though advisors will read their papers before submission, they tend to provide some feedback on paper content and limited support on language.

*“My advisor is very busy and generally does not have time to help with detailed revisions. He mainly checks whether my paper is innovative and whether the overall logic is sound. For problematic expressions, he does not directly correct them but marks them and suggests that I use some tools or editing services to make the necessary corrections. “(Interviewee 7: Doctoral student Mi)*

This is confirmed by professor Chen who shared the following points,

*“ I supervise many doctoral and master's students, so it is impractical for me to thoroughly revise each of their papers. Instead, I provide suggestions from my professional perspective, such as evaluating the logical flow from experiment to manuscript and ensuring the content arrangement aligns with academic standards. Regarding language issues, I offer general advice, but they primarily rely on translation tools and editing services for detailed corrections.” (Interviewee 2: Professor Chen)*

While seeking assistance from advisors is a common strategy among doctoral and master’s students in the field of science and engineering in China, this practice is less prevalent among university teachers and researchers. Several interrelated factors contribute to this phenomenon, including differences in academic maturity, professional expectations, the nature of research collaboration, and the established independence of faculty members.

University teachers and researchers typically possess a higher level of academic maturity and experience than their doctoral and master's student counterparts. This experience often translates into greater confidence in their writing and research abilities, reducing the perceived need for assistance from advisors. As noted by Pinheiro et al. [96], established faculty members are often expected to demonstrate a level of expertise and independence in their research endeavors, which can diminish their inclination to seek guidance.

The nature of research collaboration in academia also plays a role in this dynamic. Research by Kivlighan et al. [97] shows university teachers and researchers often engage in collaborative projects with peers, which can provide them with the necessary support and feedback without the need to seek assistance from advisors. Finally, as faculty progress in their careers, they often develop their own research agendas and writing styles, which may lead them to feel less reliant on the guidance of their advisors. This independence is supported by the findings of Sauermann & Roach [98], who note that faculty members often have established networks and resources that they can draw upon for support, further diminishing the need for advisor assistance.

#### Assistance from native English speakers.

In the interview with researcher Wu, she noted that her boss relies on a friend proficient in both native English and their field for proofreading, with expenses reimbursed. Professor Meng mentioned collaborations with experts from Europe or America, which ensure high language quality and alleviate concerns. Such opportunities, however, are rare and valuable. Lillis and Curry [4] reported that European authors preferred highly experienced native English-speaking specialists over local language teachers. Yet, “such individuals are typically not available to most scholars working in Chinese universities” [17]. For most students, encountering native English speakers with professional knowledge is rare. Even if such opportunities arise, students are unlikely to seek their help for editing without a connection through their supervisors. Consequently, there is no deliberate intention to seek native English speakers as proofreaders, leading to fewer individuals opting for this method.

#### Assistance from editing companies.

Most interviewees, except two master's students, admitted to using these services but infrequently. The use of professional editing services among students is not so widespread due to high costs. Editing services are often categorized into general, advanced, and premium levels, with higher levels being more expensive. Companies like *PaperTrue, Scribbr, Enago*, and *Editage* offer such services, with basic costs around $30 per 1,000 words. This makes it a less preferred option for students who must pay out-of-pocket. Professor Chen noted that the decision to use proofreading services depends on the target journal's prestige, suggesting it is worthwhile for high-quality journals.

Proofreading can be expensive, sometimes up to $3000, especially when figures and charts are included. Liu’s former advisor used to reimburse students for these costs, which was beneficial. According to Lim et al. [99] and interviewees in our study, authors are generally satisfied with professional editing services, though effectiveness can vary. Researcher Zhan expressed dissatisfaction, noting that edits often focus on grammar and spelling rather than coherence and logical consistency.

#### Other strategies proposed by participants.

Participants in both the open-ended questionnaire responses and interviews highlighted another effective method for improving language in writing: closely exam vocabulary, sentence structures, and expressions while reading foreign literature. During proofreading, using well-written articles as models can help identify strengths and learn from them. This approach is also recommended by previous studies, including Luo & Hyland [17] and Lin & Morison [32]. Similarly, in Cai’s study [100] on an “International Journal Paper Writing and Publication” course, students were asked to identify sentence templates in each section of published papers and build their own corpora, which proved useful for their writing.

Beyond these strategies, participants provided broader suggestions for improving English academic writing. One crucial aspect is curriculum design. Among 732 questionnaire participants, 413 had taken academic English writing courses, either as required or elective courses, or as part of a Specialized English Course; 73.61% agreed that these courses benefited their writing skills. However, interviewed students felt these courses were less helpful than expected. Most students took these courses to fulfill requirements and earn credits rather than to build skills. Additionally, these courses are typically offered during the first year of graduate studies when writing needs are not immediate, leading to a lack of motivation. By the time writing becomes crucial in later years, much of the knowledge has faded. The effectiveness of these courses is also influenced by course content, teaching methods, and student engagement. Thus, more detailed and systematic research is needed.

Participants provided valuable suggestions for curriculum implementation. For example, Professor Chen recommended specialized workshops targeting paper publication. Although the impact may not be immediate, systematic training can improve writing techniques, which is highly beneficial. Other teachers and researchers, like Liu and Wu, expressed willingness to participate in such workshops if available.

Students also suggested that universities provide online academic writing courses, allowing access to materials as needed. Additionally, sharing and discussing writing experiences in weekly seminars with advisors and fellow students can aid in writing papers for publication.

## Conclusions

### Summary of the results and findings

This study identifies several linguistic challenges faced by Chinese academics in science and engineering when writing for English-language scholarly publication. Sentence construction emerged as the most significant challenge across all academic groups. This highlights the complexity of adapting to English syntactic structures, especially for non-native speakers accustomed to Chinese linguistic patterns. Vocabulary selection and coherence are also prominent challenges, influenced by limited academic vocabulary, unfamiliarity with nuanced word usage, and the negative transfer of native language conventions. Grammar, while still a concern, was ranked as the least challenging linguistic aspect, particularly among more experienced academics. Notably, master's students perceived grammar as significantly greater challenges compared to doctoral students and university teachers or researchers.

The study also highlights various strategies employed to mitigate these challenges. Online platforms and software tools were the most commonly used resources, particularly among master's students. Tools like Grammarly and machine translation services were frequently utilized for pre-editing and proofreading. Peer support from proficient English speakers within the academic community was a widely adopted strategy, especially among students. Doctoral students heavily relied on advisors for guidance and feedback, though the level of language-specific assistance varied. Professional editing companies were used selectively, primarily for submissions to high-impact journals, due to their high costs. Assistance from native English speakers was less commonly utilized due to limited accessibility and connections.

These findings contribute to the broader discourse on linguistic barriers in academic publishing and the inequities faced by non-native English-speaking scholars. They underscore the need for institutional support, such as tailored academic writing programs, access to language resources, and mentorship opportunities, to enhance the global accessibility and inclusivity of scholarly communication.

### Implications, contribution, and value of the study

#### Implications for practice and policy.

1. Enhanced academic writing training:

Universities should establish comprehensive academic writing courses tailored to address the specific needs of non-native English speakers.

 Workshops on leveraging advanced tools like AI-based grammar checkers and translation software can empower authors to refine their work independently.

2. Institutional support systems: 

 Universities and research institutions should subsidize professional editing services or provide access to native English-speaking editors for early-career researchers and students.

 Peer mentoring programs where experienced academics guide younger scholars through the publication process could significantly improve writing quality and confidence.

3. Cross-disciplinary collaboration:

 Promoting collaborations with international researchers can enhance language proficiency through exposure to best practices in academic writing and foster greater global visibility for Chinese scholars.

4. Integration of technological tools:

 Encouraging the use of AI-powered writing tools like Grammarly, ChatGPT, and other advanced platforms could assist non-native authors in overcoming basic linguistic barriers.

5. Policy Recommendations:

 National funding bodies should consider supporting editing and language refinement initiatives, particularly for young researchers aiming to publish in high-impact international journals.

#### Contribution of the study.

This study specifically investigates the linguistic challenges faced by Chinese academics in science and engineering, which provides a comparative analysis across academic ranks (university teachers and researchers, doctoral students, and master's students), offering nuanced insights into varying perceptions and coping strategies.By integrating quantitative data from 732 questionnaire responses with qualitative insights from 13 interviewees, the study ensures a robust and triangulated understanding of the issues. The mixed-methods approach highlights both the common and unique challenges faced by different academic groups, adding depth to the analysis.

The study provides actionable suggestions for individuals, institutions, and policymakers, making its findings directly applicable to improving the academic writing process and publication success rates. It also emphasizes the role of online platforms, translation tools, and professional editing services, offering insights into how technology can complement traditional writing practices.

#### Limitations.

Despite the strengths of combining quantitative and qualitative approaches, this study has some limitations. First, while it systematically identified the challenges faced by academics and incorporated insights from writing instructors and journal reviewers, it did not directly compare these reported challenges with actual manuscripts written by the participants. Therefore, Future research could conduct comparative analyses between author's perceptions and their written work to provide a more comprehensive understanding.

Second, though challenges and strategies were identified and summarized, more detailed and separate research on coping strategies can be conducted. For example, the study did not separately examine coping strategies for mastering lengthy scientific and engineering terminology. Future studies could explore discipline-specific lexical challenges in greater depth, which would offer more targeted recommendations for academic writing support.

Third, the focus on Chinese academics in science and engineering may limit the generalizability of the findings to other disciplines or cultural contexts. Expanding future research to include a broader range of fields and academic backgrounds would enhance the applicability of the results.

Finally, this study provides a cross-sectional view without tracking participants’ development over time. Longitudinal studies would be valuable to capture the evolution of writing skills and the long-term effectiveness of coping strategies.

## Supporting information

S1 File Inclusivity in global research questionnaire. (DOC)

S2 File Questionnaire. (DOC)

S3 File Interview questions. (DOC)

S4 Table Raw data of tables. (XLS)
