# Supplementary material for: Linguistic challenges of writing papers in English for scholarly publication: Perceptions of Chinese academics in science and engineering
Source: PLoS One. 2025 May 27;20(5):e0324760. doi: 10.1371/journal.pone.0324760 (PMC12111667; doi:10.1371/journal.pone.0324760)
Supplement: S1 File — (DOC) [file pone.0324760.s001.doc]

# Inclusivity in global research

PLOS’ policy on inclusivity in global research aims to improve transparency in the reporting of research performed outside of researchers’ own country or community and ensures that PLOS publications reporting global research adhere to high standards for research ethics and authorship. Authors of relevant research articles may be asked to complete the questionnaire below, which outlines ethical, cultural, and scientific considerations specific to inclusivity in global research. This questionnaire may be requested when researchers have travelled to a different country to conduct research, if research uses samples collected in another country, research with Indigenous populations or their lands, or if research is on cultural artefacts. Researchers travelling to another country solely to use laboratory equipment will not normally be required to complete the questionnaire. However, the questionnaire can be requested at the journal’s discretion for any submission – if you have been requested to complete this questionnaire by the PLOS journal you submitted to, please do so.

Please complete the questionnaire below and include this as a Supporting Information file with your manuscript. Note that if your paper is accepted for publication, this checklist will be published with your article in the supporting information files. Please ensure that you reference the checklist in the main body of your manuscript. We suggest adding a subsection ‘Inclusivity in global research’ to your Methods section and adding the following sentence: “Additional information regarding the ethical, cultural, and scientific considerations specific to inclusivity in global research is included in the Supporting Information (SX Checklist)”

The questions have been designed to be applicable to a wide range of study types, and there are subsections for both human subjects research and non-human subjects research. If any of the questions are not relevant to your research please mark them as “N/A” as appropriate.

**Ethical considerations, permits and authorship**

*This section is applicable to all research types.*

Provide details as to who granted permissions and/or consent for the study to take place in the Methods section of your manuscript. This should include the names of **all** ethics boards, governmental organizations, community leaders or other bodies that provided approval for the study. If individuals provided approval refer to these people by their role or title but do not list their name(s).

Reported on page number: P. 6. The research protocol was reviewed and approved by the Ethics Committee of Graduate School of International Cultural Studies, Tohoku University, with the approval number: 2023-13.

If there were any deviations from the study protocol after approval was obtained please provide details of these changes in the Methods section of your manuscript.
Did this study involve local collaborators that are residents of the country where the research was conducted or members of the community studied? If you do not have any authors from said communities, please provide an explanation for this below.

Reported on page number: N/A

Yes, this study involved local collaborators who are residents of the country where the research was conducted and/or members of the community studied. Their contributions were integral to the research process, particularly in ensuring cultural relevance, ethical appropriateness, and contextual understanding of the study.

Everyone listed as an author should meet PLOS’ criteria for authorship and all individuals who meet these criteria should be included in the author byline, rather than the acknowledgements. For further information please see the journal’s Authorship Policy.

**Human subjects research (e.g. health research, medical research, cross-cultural psychology)**

Did you obtain written informed consent from a representative of the local community or region before the research took place? How did you establish who speaks for the community? Details of written informed consent obtained from study participants should be reported separately in the Methods section of your manuscript.

All participants were fully informed about the nature and purpose of the study. Written informed consent was obtained from each participant prior to their involvement in the study. Participants in the questionnaire survey must check the option agreeing to participate in the survey before they can proceed with the questionnaire. Therefore, all the valid questionnaires received are from participants who have given their consent. All thirteen interviewees provided signed consent to participate.

The consent provided participants with detailed information about the study's objectives, procedures, potential risks, and benefits. Participants were assured of the confidentiality and anonymity of their responses. They were informed that participation was voluntary and that they could withdraw from the study at any time without any negative consequences. The information is included in Methods section. The signed written consent is uploaded as supplement materials.

How did members of the local community provide input on the aims of the research investigation, its methodology, and its anticipated outcome(s)?

We took several measures to ensure that the informed consent documents and other materials were understandable by local stakeholders:

**Translation and Localization:** All documents were translated into the local language(s) spoken by the community. The translations were reviewed by native speakers and cultural experts to ensure accuracy and appropriateness.

**Simplified Language:** The language used in the materials was simplified to avoid technical jargon and ensure accessibility to individuals with varying levels of education.

**Cultural Sensitivity:** We worked with local collaborators or cultural mediators to ensure the materials were culturally sensitive and aligned with local customs and norms.

**Pre-Testing Materials:** Draft versions of the materials were shared with a small group of local stakeholders for feedback, and revisions were made based on their suggestions.

**Verbal Explanations:** In addition to providing written documents, we conducted face-to-face or virtual sessions to explain the content verbally, allowing stakeholders to ask questions and seek clarifications.

**Ongoing Support:** We ensured that stakeholders had ongoing access to support and could ask questions or raise concerns throughout the research process.

When engaging with the local community, how did you ensure that the informed consent documents and other materials could be understood by local stakeholders

Questionnaire, interviews were translated and conducted in the local language(Chinese) spoken by the community. The translations were reviewed by native speakers and cultural experts to ensure accuracy and appropriateness.

**Pre-Testing Materials:** Draft versions of the materials were shared with a small group of local stakeholders for feedback, and revisions were made based on their suggestions.

**Verbal Explanations:** In addition to providing written documents, we conducted face-to-face or virtual sessions to explain the content verbally before interviews, allowing stakeholders to ask questions and seek clarifications.

**Ongoing Support:** We ensured that stakeholders had ongoing access to support and could ask questions or raise concerns throughout the research process

Yes, the findings of the research will be made available to stakeholders in the community in an accessible and understandable format. We plan to achieve this through the following methods:

**Summary Report:** A concise and easy-to-understand summary report will be prepared, highlighting the key findings and their implications. This report will be written in clear, non-technical language and translated into the local language(s) if necessary

**Copies of Publications:** Copies of any publications or reports arising from the research will be made available to local stakeholders, either in print or electronically, depending on their preferences and accessibility.

**Direct Communication:** For participants who expressed interest during the interviews or surveys, we will follow up with personalized communication to share the findings, such as email summaries or printed copies delivered locally.

These steps are intended to ensure that the community benefits from the research outcomes and that the findings are both accessible and meaningful to them

Will the findings of the research be made available in an understandable format to stakeholders in the community where the study was conducted (e.g. via a presentation, summary report, copies of publications, etc.)? Please provide details of how this will be achieved.

Yes, the findings of this research will be made available to stakeholders in the community where the study was conducted in a format that is both understandable and accessible. This will be achieved through the following methods:

Summary Report:

A concise and reader-friendly summary report will be prepared, highlighting the key findings and their implications. This report will avoid technical jargon and, if needed, will be translated into the stakeholders’ primary language to ensure comprehensibility.

Copies of Publications:

Copies of any published papers arising from the research will be shared with stakeholders. These will be provided in both digital and printed formats, depending on the stakeholders' preferences and accessibility requirements.

Direct Communication:

For participants who expressed interest in the research findings during data collection, personalized emails or printed summaries will be sent directly. This ensures that participants receive tailored communication of the outcomes.

**Non-human subjects research using specimens/ animals collected as part of the study, or those housed in archival collections. Examples include archaeology, paleontology, botany and zoology.**

Did the permission you obtained from a local authority to perform the study include an agreement on access to outputs and benefit sharing? This may include procedures to enable fair distribution of the benefits and resources arising from the research performed. Please include any details of Prior Informed Consent and Benefit Sharing Agreements obtained. These may be required by field-specific regulations, for example the Convention on Biological Diversity (CBD) and the associated Nagoya Protocol.

N/A

If the material used in your study was imported, please A) provide the year it was imported and B) indicate whether permits were obtained to import/export the materials used, C) provide details of any permits obtained. If this information is not available, please indicate this.

N/A

If you used archival specimens, please state how the material used in your study was acquired by the institute it is held in and provide details of any permits obtained for the original excavations/ sample collection. If this information is not available, please indicate this.

N/A

How was the potential cultural significance of the materials collected in your study to local communities considered in your research design? Were Indigenous peoples and/or local researchers and institutions involved with archaeological excavations / collection of specimens? If so, please provide a description of their involvement.

N/A

If your manuscript includes photographs of human remains please indicate whether authors obtained permission from descendants or affiliated cultural communities to do so.

N/A
