# Supplementary material for: Linguistic challenges of writing papers in English for scholarly publication: Perceptions of Chinese academics in science and engineering
Source: PLoS One. 2025 May 27;20(5):e0324760. doi: 10.1371/journal.pone.0324760 (PMC12111667; doi:10.1371/journal.pone.0324760)
Supplement: S2 File — (DOC) [file pone.0324760.s002.doc]

Questionnaire

Linguistic Challenges of Writing Papers in English for Scholarly Publication: Perceptions of Chinese Academics in Science and Engineering

**Section 1: Basic information**

1) Your university or institute ( )

2) Your identity

□ University teacher □ Teaching Assistant □ Lecturer

□ Associate Professor □ Professor

□ Researcher □ Assistant Researcher

□ Associate Researcher£ Researcher

□ Student □ Master's student □ Doctoral student (Which year: )

3) Your field of study ( )

4) Your native language (　　　　　　　　)

**Section 2: Your attitude toward writing papers in English and experience of publication**

5) Writing papers in English is important for your career or study.

□ Strongly agree □ Agree □ Somewhat agree

□ Somewhat disagree □ Disagree □ Strongly disagree

1. English is important for paper writing for scholarly publication in international journals.

□ Strongly agree □ Agree □ Somewhat agree

□ Somewhat disagree □ Disagree □ Strongly disagree

7) Have you ever published papers in English?

□ Yes □ No

**Section 3: Linguistic challenges of writing in English for scholarly publication.**

8) Using correct grammar is challenging.

□ Strongly agree □ Agree □ Somewhat agree

□ Somewhat disagree □ Disagree □ Strongly disagree

9) Using proper words is challenging.

□ Strongly agree □ Agree □ Somewhat agree

□ Somewhat disagree □ Disagree □ Strongly disagree

10) Constructing proper sentences is challenging.

□ Strongly agree □ Agree □ Somewhat agree

□ Somewhat disagree □ Disagree □ Strongly disagree

11) Using proper cohesive devices is challenging.

□ Strongly agree □ Agree □ Somewhat agree

□ Somewhat disagree □ Disagree □ Strongly disagree

12) Organizing content with coherence is challenging.

□ Strongly agree □ Agree □ Somewhat agree

□ Somewhat disagree □ Disagree □ Strongly disagree

13) Other challenging aspects: ( )

**Section 4: Strategies of overcoming linguistic challenges when writing papers in English.**

14) You use online platforms /software tools.

□ Strongly agree □ Agree □ Somewhat agree

□ Somewhat disagree □ Disagree □ Strongly disagree

15) You seek assistance from your advisor.

□ Strongly agree □ Agree □ Somewhat agree

□ Somewhat disagree □ Disagree □ Strongly disagree

16) You seek assistance from native Chinese speakers who have good command of English.

□ Strongly agree □ Agree □ Somewhat agree

□ Somewhat disagree □ Disagree □ Strongly disagree

17) You seek assistance from native English speakers.

□ Strongly agree □ Agree □ Somewhat agree

□ Somewhat disagree □ Disagree □ Strongly disagree

18) You seek assistance from professional editing companies.

□ Strongly agree □ Agree □ Somewhat agree

□ Somewhat disagree □ Disagree □ Strongly disagree

19) If you have ever sought assistance from professional editing companies, which price range does it belong to?

100~200 dollars

201~300 dollars

301~400 dollars

401~500 dollars

501~600 dollars

Other price ranges: ( )

20) Other strategies you use to overcome linguistic challenges when writing papers in English: ( )

**Section 5: Your perceptions of English academic writing curriculum.**

21) Have you ever taken or are you taking an English academic writing course?

□ Yes □ No

22) You think English academic writing is beneficial to your writing papers in English for scholarly publication.

□ Strongly agree □ Agree □ Somewhat agree

□ Somewhat disagree □ Disagree □ Strongly disagree

23) Besides English academic writing course, have you ever resorted to following resources to improve your English academic writing ability. (Select all that apply)

□ Online English academic course

□ Face to face training course

□ Lectures

□ Books

Other resources: ( )

24) Do you have other specific opinion regarding challenges and strategies of writing papers in English for scholarly publication. (Open-ended question)
