# Supplementary material for: Linguistic challenges of writing papers in English for scholarly publication: Perceptions of Chinese academics in science and engineering
Source: PLoS One. 2025 May 27;20(5):e0324760. doi: 10.1371/journal.pone.0324760 (PMC12111667; doi:10.1371/journal.pone.0324760)
Supplement: S3 File — (DOC) [file pone.0324760.s003.doc]

Interview questions

1) Is writing papers in English important for your career or study? Why is it important and how does it relate to your career and study?

2) Besides the content of the paper, do you think that English language is important? Please specify your reasons.

3) Do you think that writing papers in English is difficult?

4) Have you ever published papers in English?

5) Do you have any experience of paper rejection? If yes, what are the specific reasons that lead to rejection?

6) What are challenging aspects of your writing papers in English? Why these aspects are challenging for you? Can you specify these aspects with examples?

7) As an English academic writing course instructor, what are your students’ problems of papers in English? (Optional according the identity of interviewees)

8) As a supervisor, what are your students’ problems of writing papers in English? (Optional according the identity of interviewees)

9) What strategies do you use to overcome linguistic challenges when you write papers in English? Why do you think they are helpful? Can you specify with examples?

10) Do you have other specific opinion regarding challenges and strategies of writing papers in English for scholarly publication?
